# Acute Angle-Closure Glaucoma as an Ocular Complication of Dengue Fever: A Comprehensive Review

**DOI:** 10.7759/cureus.82119

**Published:** 2025-04-11

**Authors:** Aiad Al-Essa

**Affiliations:** 1 Department of Ophthalmology, Maharishi Markandeshwar University, Solan, IND

**Keywords:** acute angle-closure glaucoma, clinical intervention, dengue fever, diagnostic imaging, intraocular pressure, nd:yag iridotomy, ocular complications

## Abstract

Dengue fever, a rapidly spreading global epidemic, ranks among the most frequently reported viral infections worldwide, typically presenting with systemic symptoms such as fever, headache, and rash. Although generally linked to systemic issues, dengue infection can also lead to uncommon ocular complications, such as acute angle-closure glaucoma (AACG), which poses a threat to sight. This review focuses on the alterations in the anterior chamber angle and the subsequent development of glaucoma after dengue infection. Prompt and precise diagnosis, aided by essential diagnostic tools like intraocular pressure (IOP) measurement, gonioscopy, and advanced imaging methods, is essential for effective management. The initial treatment typically includes osmotic agents, carbonic anhydrase inhibitors, and topical glaucoma medications, with Nd:YAG (neodymium-doped yttrium aluminum garnet) laser iridotomy being the definitive treatment option. The review emphasizes the importance of long-term monitoring to assess ocular outcomes and prevent relapse. While AACG occurrence in dengue patients is rare, healthcare providers in endemic areas must stay alert for ocular symptoms in dengue patients presenting with vision problems. Preventing irreversible vision loss demands increased vigilance, early diagnosis, and timely treatment. Additionally, the review advocates for more clinical research and the creation of refined diagnostic protocols to tackle this rare but severe complication.

## Introduction and background

Dengue fever is a major global public health concern that is quickly expanding geographically and becoming more clinically complex. This mosquito-borne viral disease, mostly transmitted by *Aedes aegypti*, has rapidly spread throughout tropical and subtropical countries as a result of increased urbanization, worldwide travel, and climate change [1,2]. The disease is caused by one of four dengue virus serotypes (DENV-1 to DENV-4) from the *Flavivirus* family, with an estimated annual incidence of 390 million [3,4].

Dengue is classically a febrile illness with headache, retro-orbital pain, muscle and joint aches, rash, and leukopenia. More severe forms, such as dengue hemorrhagic fever (DHF) and dengue shock syndrome (DSS), present with features of increased vascular permeability, thrombocytopenia, and a bleeding tendency [5,6]. Recent studies have also highlighted the neurological, cardiac, and ophthalmic complications, and have pointed out the multisystemic manifestations of dengue [7,8].

Ophthalmic complications are among the lesser-known, clinically significant manifestations. Reports detail various conditions, ranging from subconjunctival hemorrhage and anterior uveitis to severe optic neuritis, retinal vasculitis, maculopathy, and panophthalmitis [9-11]. Other diverse ocular effects include central retinal artery occlusion and bilateral vitreous hemorrhage [12,13].

A small but significant number of studies have found acute angle-closure glaucoma (AACG) in dengue-infected patients, indicating that inflammation might cause ocular structural alterations. Angle closure in the anterior chamber causes an emergent ophthalmologic condition known as AACG, which is a blockage of aqueous humor outflow. This can also result in a very rapid rise in intraocular pressure (IOP), leading to immediate visual disturbances, pain, nausea, and potential vision loss [14-16]. While the majority of AACG cases result from anatomical predispositions and pharmacologic triggers, infections - including dengue - have been considered secondary causes, owing to inflammatory and vascular permeability changes [17,18].

**Figure 1:**
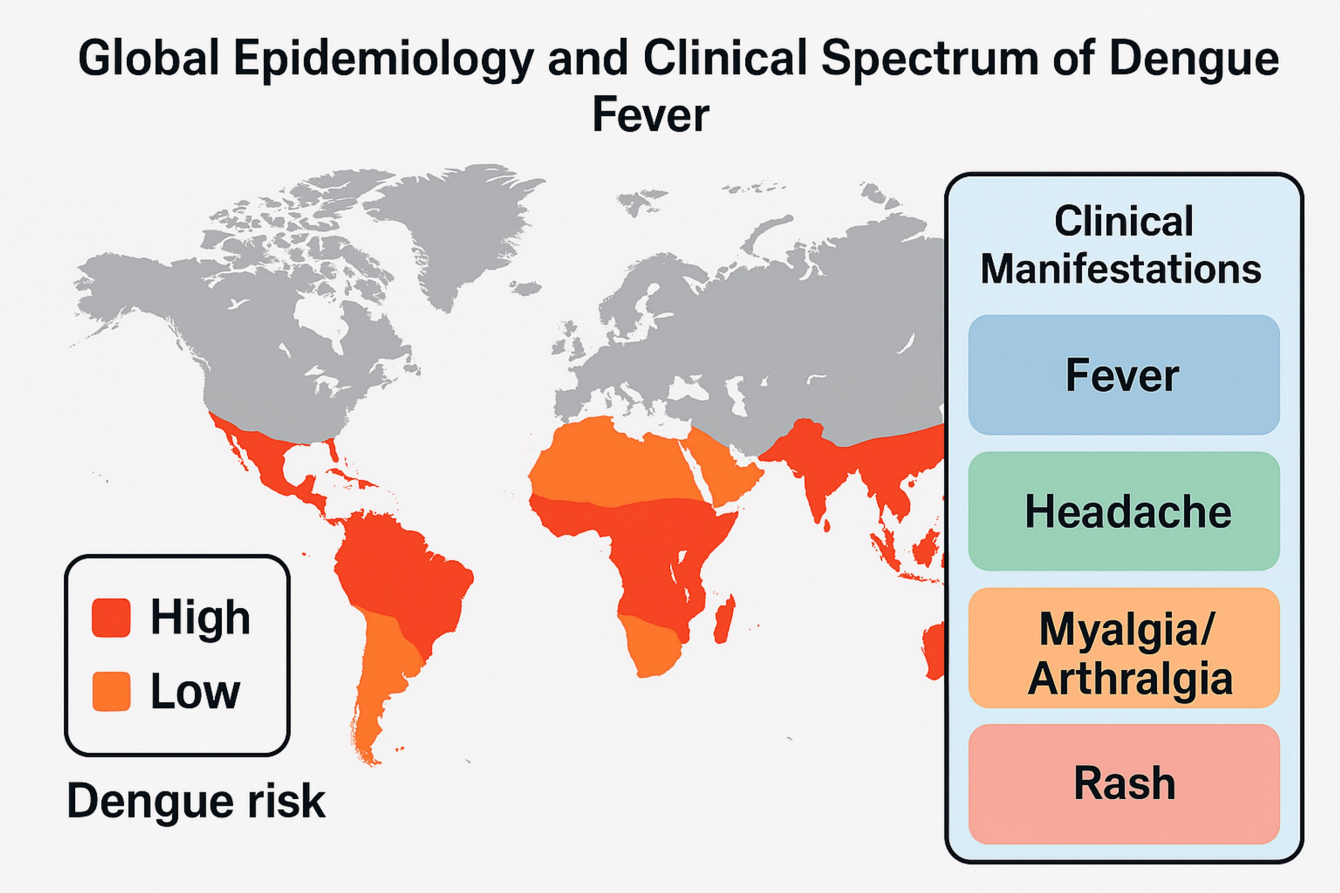
Global Epidemiology and Clinical Spectrum of Dengue Fever Image credit: Created using Datawrapper (https://www.datawrapper.de/)

Mainstream literature underreports the potential association between dengue fever and AACG. Clinical studies and case series, however, suggest a plausible pathophysiological link, including ciliochoroidal effusion, forward displacement of the lens-iris diaphragm, and immune-mediated angle narrowing [19,20]. Additional support that the dengue virus is capable of causing ocular structural and functional disruption comes from ophthalmic literature on viral hemorrhagic fevers and immune-mediated uveitis [21,22]. Figure 1 presents the epidemiologic reach and clinical diversity of dengue as a global burden and an expanding clinical spectrum.

This review seeks to bridge that gap by synthesizing clinical, pathological, and diagnostic insights from a single representative case of dengue-induced AACG. We place this in the framework of the wider epidemiology of dengue and review the existing literature on ocular complications of viral illnesses and various plausible mechanisms. We also take into consideration systemic factors - i.e., autoimmune responses, drug-induced exacerbations, and concomitant diseases - that can add to the clinical picture [23,24].

Objectives of the review

This review aims to explore ocular pathology associated with dengue fever, with a specific emphasis on AACG. It seeks to thoroughly describe the clinical manifestations, as well as the diagnostic and therapeutic management approaches regarding dengue fever-associated AACG. In addition, this review attempts to define key diagnostic considerations, assess a series of treatment outcomes, and discuss wider clinical implications through a case study. The purpose of the review is to highlight these aspects so that clinicians will be aware of this rare but severe ophthalmologic complication associated with dengue, and can therefore recognize it early, treat it promptly, and reduce the risk of visual impairment.

## Review

Clinical presentation and diagnostic challenges

Initial Systemic Presentation of Dengue

Dengue fever is usually characterized by a sudden onset of high fever and prominent symptoms, including headache, generalized weakness, muscle and joint pain, and marked fatigue [1,9]. Notable retro-orbital pain is a common occurrence in patients, and this pain further increases the discomfort and intensity of symptoms [25]. The febrile phase usually lasts two to seven days, and there may also be additional symptoms, such as gastrointestinal upset, nausea, vomiting, and occasionally, abdominal pain [3,4]. Sometimes, the presence of rash and signs of bleeding may alert clinicians to more severe forms of dengue. Early recognition of the systemic manifestations of dengue is essential, especially in endemic areas, since prompt treatment can minimize morbidity and prevent DHF or DSS [5].

Emergence and Characterization of Ocular Symptoms

Ocular complications in dengue are relatively uncommon but clinically significant. The manifestations vary from mild conjunctival congestion and subconjunctival hemorrhage to severe retinal hemorrhage, uveitis, optic neuropathy, and macular edema [13,26]. The visual symptoms may be isolated or associated with systemic features, which may delay diagnosis. AACG is one of the severe ocular conditions associated with dengue, characterized by intense ocular pain, sudden loss or blurring of vision, photophobia, perception of halos around lights, severe headache, and nausea [14,15]. Given these findings, the patient should be urgently evaluated by an ophthalmologist and undergo immediate intervention to control IOP to prevent irreversible damage [27]. Early suspicion and swift action are crucial because the window is narrow for therapeutic response.

Early Detection and Differential Diagnosis

Dengue fever, being a rapid, progressive infection with a risk of irreversibly damaging the optic nerve, makes prompt detection and correct diagnosis of AACG of paramount importance. The most common cause of AACG is acute anatomical blockage of aqueous humor drainage, usually due to anterior displacement of the iris, resulting in a shallow anterior chamber [20]. A significantly worsened prognosis can occur due to misdiagnosis or delayed intervention. Other causes of acute ocular pain and vision impairment should be considered in the differential diagnosis of AACG, including conditions such as anterior uveitis, conjunctivitis, corneal abrasion, or secondary glaucoma, induced by systemic illness or medications [28,29]. A careful ophthalmological examination helps to distinguish AACG from these conditions.

These include visual acuity examination, slit-lamp biomicroscopy, measurement of IOP through tonometry, evaluation of angle closure using gonioscopy, as well as advanced imaging - for example, anterior segment optical coherence tomography (AS-OCT) [30]. These evaluations permit precise assessment of anterior chamber depth, angle status, and confirmation of the glaucomatous process. In the clinical setting of dengue, a high index of suspicion for ocular symptoms is essential. Bedside clinical assessments, when performed at the right time, facilitate early recognition and appropriate intervention, which may prevent permanent visual impairment.

The differential diagnosis of AACG associated with dengue is thus an important practice, especially in recognizing these ocular symptoms early on. These practices have a major impact on patient outcomes and the preservation of visual function. A chronological view of the symptom progression and major diagnostic markers in a representative case is provided in Table 1.

**Table 1:** Timeline of Clinical Presentation and Key Diagnostic Events Source: This table was constructed by synthesizing clinical patterns reported in previously published literature on dengue-associated ocular complications and AACG management [5,14,19,20]. AACG: Acute Angle-Closure Glaucoma; AS-OCT: Anterior Segment Optical Coherence Tomography

Day	Clinical Presentation and Diagnostic Events
1	Onset of high-grade fever, severe headache, generalized weakness
2	Continued fever, onset of ocular symptoms (pain, redness, photophobia)
3	Hospital admission, initial ophthalmologic evaluation (visual acuity, tonometry)
4	Gonioscopy and AS-OCT confirm shallow anterior chambers and acute angle closure.
5	Initiation of immediate ocular management (mannitol, topical antiglaucoma therapy)
6	Improvement in ocular symptoms, decreased intraocular pressure.
7	Stabilization of ocular condition, improvement in visual acuity

Pathophysiology and mechanisms

Dengue Virus and Immune-Mediated Inflammatory Response

Immune and inflammatory responses that can affect many organ systems, including the eye, are induced in the course of dengue virus infection. After the virus enters the host, dendritic cells present the viral antigens, activate CD4+ and CD8+ T cells, release cytokines, and activate complement [28]. Although critical for viral clearance, these responses also lead to systemic and local inflammation. The phenomenon of antibody-dependent enhancement (ADE) may worsen immune dysregulation further and increase vascular permeability during secondary infections [4]. Widespread endothelial dysfunction is a central pathological mechanism in dengue-related complications due to the overproduction of inflammatory mediators, such as tumor necrosis factor-alpha (TNF-α), interleukin-6 (IL-6), and interferon-gamma (IFN-γ). Thus, these immune dysregulations induce immune injury to the blood-ocular barrier and increase the eye’s vulnerability to inflammatory injury.

Vascular Leakage and Ocular Implications

Plasma leakage into interstitial and tissue compartments is one of the hallmark features of severe dengue, characterized by increased vascular permeability. This permeability may occur in the ocular system, with retinal hemorrhages, macular edema, and choroidal effusions [26]. The ocular structures have a particularly poor tolerance for inflammatory insult and disruption of vessel regulation due to their rich choroid and ciliary body vasculature. These structures are involved in vascular leakage, which causes forward displacement of the lens-iris diaphragm and narrowing of the anterior chamber angle, potentially leading to AACG [9]. Edema in the ciliary body exacerbates the mechanical crowding of the angle structures, increasing outflow resistance. When IOP rises rapidly because of poor aqueous outflow, the optic nerve becomes vulnerable to ischemic injury.

Inflammatory Edema and Anterior Chamber Alterations

Dengue can directly inflame ocular anatomical tissues. The iris-lens diaphragm might rotate and move forward due to ciliary body edema, resulting in shallowing of the anterior chamber [20]. This type of anatomical change impedes aqueous fluid outflow through the trabecular meshwork, which, in predisposed individuals, may lead to a sudden rise in IOP. In addition, the inflammatory response may lead to trabeculitis, which increases resistance in the drainage pathways. Timely ophthalmologic intervention is important in such cases because the dynamic changes in ocular fluid dynamics under inflammatory stress are highlighted. Early diagnosis may be missed, as these changes may be subtle at first, and without proper ophthalmologic tools, such as gonioscopy and AS-OCT.

Drug-Induced and Systemic Interactions

Systemic medications and dengue-associated systemic effects that may affect ocular physiology should also be considered. AACG can be precipitated by certain drugs used to treat dengue complications, or by medications that patients may have been taking before hospitalization. For example, sulfonamide-based antibiotics and anti-epileptics, such as topiramate, have been associated with drug-induced AACG due to ciliochoroidal effusion and angle closure [14,15]. Severe dengue may cause systemic capillary leak syndrome, which acts synergistically with these pharmacologic triggers to worsen anterior segment crowding and precipitate glaucoma. In addition, systemic hypovolemia and electrolyte imbalances may exacerbate vascular instability in ocular tissues, leading to fluid extravasation and segmental edema.

Autoimmune Pathways and Molecular Mimicry

Molecular mimicry has been implicated as the mechanism leading to viral infections, causing the triggering of autoimmune reactions. The concept relates to the structural similarity between the viral peptides and host ocular antigens, so that ocular tissues can be auto-targeted [18]. While dengue is less explored in terms of the disease process in the anterior segment, parallels between other flavivirus infections suggest that dengue may have an associated autoimmune-mediated inflammation in the anterior segment. Such immune responses would further augment ciliary body edema, interfere with aqueous production and outflow, and have detrimental effects on IOP regulation. In addition, genetic predispositions associated with human leukocyte antigen (HLA) alleles could participate in the autoimmune eye inflammation induced by the dengue virus and need to be further studied.

Hypothesized Sequence of Events

**Figure 2:**
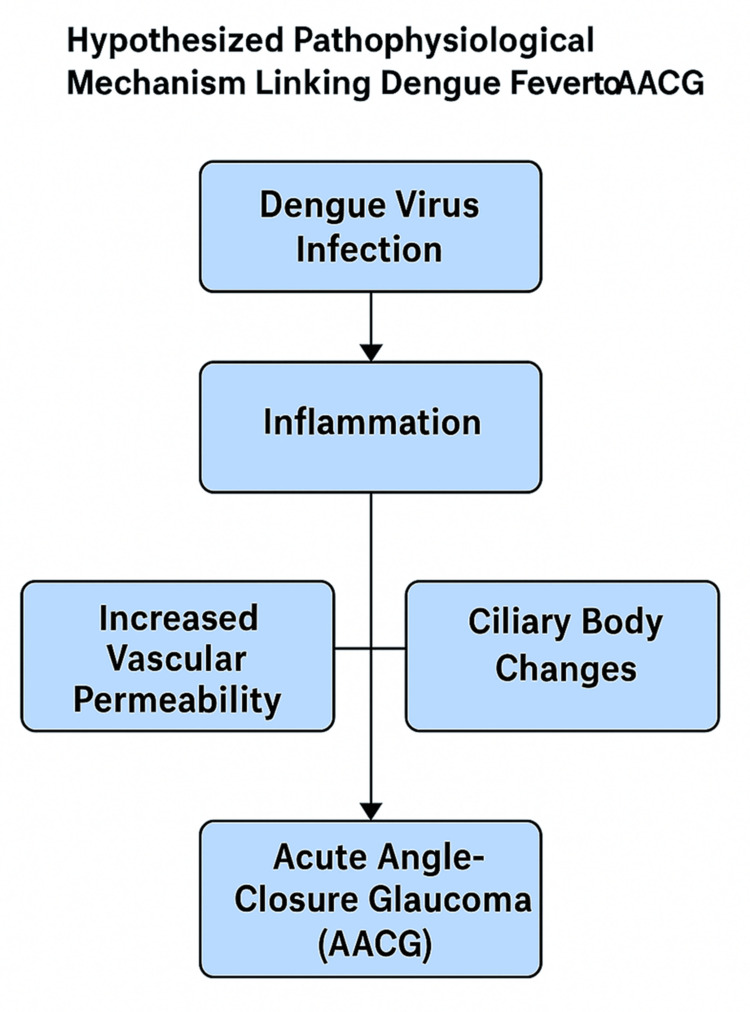
Hypothesized Pathophysiological Mechanism Linking Dengue Fever to AACG AACG: Acute Angle-Closure Glaucoma

Dengue exhibits a wide spectrum of systemic and localized immune responses, and considering this, we can hypothesize a sequence in which dengue virus infection generates a systemic inflammatory response mediated by cytokines. This, in turn, leads to vascular permeability and ciliary body extravasation of fluid, primarily resulting in ocular edema. The resulting anterior segment anatomical changes lead to narrowing or closure of the anterior chamber angle, which ultimately leads to AACG. Additionally, the dengue pathophysiology, medications, individual anatomical predispositions, and possibly autoimmune pathways provide a multifactorial basis for AACG development in dengue-affected individuals. Figure 2 depicts the suggested sequence of pathophysiological events from dengue fever to AACG.

Diagnosis and clinical findings

Comprehensive Ocular Examination Protocols

A meticulous, multi-step ophthalmologic evaluation is required to accurately diagnose AACG, especially in patients with systemic infections such as dengue. The first step usually involves a review of all symptoms and medical history, followed by a basic ocular examination, such as visual acuity testing. Patients often present with sudden vision loss, halos around lights, or problems seeing clearly [5,31], due to general difficulty with vision and visual acuity caused by corneal edema and optic nerve compromise in AACG [1]. On external inspection, conjunctival injection, a hazy cornea, and a mid-dilated, nonreactive pupil may be seen.

IOP measurement is an integral part of a diagnostic test. Typically, AACG causes a rapid elevation in IOP >40 mmHg, and ischemic optic neuropathy can occur in the absence of compromise of the retrolental space [14]. Handheld or rebound tonometers are useful in acute or non-clinical settings and are considered the gold standard compared to Goldmann applanation tonometry. Immediate initiation of pressure-lowering therapies is necessary when elevated IOP is recognized [19].

Anterior chamber structures are assessed by slit-lamp biomicroscopy, corneal edema is detected, and inflammatory cells or flares in the aqueous humor are observed. A shallow anterior chamber and iris bombé may suggest pupillary block mechanisms, while flare and cells may suggest coexisting uveitis, both of which are recognized dengue-related ocular complications [9,22].

Diagnostic Imaging and Gonioscopic Observations

AACG remains the most definitive diagnostic tool to confirm a given diagnosis of AACG. Clinicians can directly visualize the iridocorneal angle and determine whether the angle is closed, occludable, or open. In AACG, the angle is usually closed completely, with peripheral anterior synechiae (PAS) in chronic or recurrent cases. The diagnosis is supported by the inability to view the trabecular meshwork without indentation gonioscopy [20].

Imaging of anterior segment anatomy (gonioscopic, AS-OCT, or ultrasonic biomicroscopy (UBM)) might enhance gonioscopic findings (offering data on the anatomic structure, which may be masked by the pupil dose or obscured by the lens) and provide non-invasive data about the anterior segment. The angle structure can be delineated, and parameters such as angle opening distance and trabecular-iris space area can be measured with AS-OCT. These measurements are important to confirm angle closure, especially in eyes with a corneal haze that prevents direct gonioscopic visualization [29,30].

Deeper penetration is provided by UBM, which visualizes the ciliary body and posterior chamber structures. It has been particularly useful in detecting ciliochoroidal effusion, which is suspected to play a role in the forward rotation of the ciliary body and angle narrowing in dengue-associated AACG [27,32].

Clinical Significance of Axial Length and Anterior Chamber Depth

Anatomical predispositions to AACG are best understood in terms of biometric measurements. Like most ocular diseases, primary angle closure disease is related to a short axial length (generally less than 22 mm) and decreased anterior chamber depth. A-scan ultrasonography or optical biometry can be used to assess these parameters [24].

These predisposing factors may be further exacerbated in dengue patients by disease-induced changes, such as increased vascular permeability and inflammatory edema. Other studies have shown that dengue infection can cause choroidal thickening or ciliary body swelling, which pushes the lens-iris diaphragm forward, further narrowing the angle of the anterior chamber [17,18].

Axial length and anterior chamber depth are notably useful for both diagnostic clarity and critical long-term planning. Surgical options, such as lens extraction or filtering procedures, may be considered when recurrent or refractory cases, along with biometric findings, confirm high-risk anatomy [16].

Systemic Integration and Follow-Up

Systemic and ophthalmic overlap make it imperative that such assessments are interdisciplinary. Systemic symptoms and ocular emergencies need to be managed concomitantly by multiple departments, including internal medicine, infectious disease, and ophthalmology. Periodic IOP checks, optic nerve imaging, and visual field assessment should be performed as follow-up to monitor for glaucoma progression [6,21]. Table 2 provides a comprehensive summary of diagnostic findings, including visual acuity, IOP, and imaging results.

**Table 2:** Summary of Diagnostic Findings AS-OCT: Anterior Segment Optical Coherence Tomography; UBM: Ultrasound Bio-Microscopy; AC: Anterior Chamber

Diagnostic Component	Key Findings	References
Visual Acuity	Markedly reduced, often to counting fingers or worse	[31]
Intraocular Pressure	Elevated (>40 mmHg)	[14,19]
Slit-Lamp Examination	Shallow anterior chamber, corneal edema, iris bombé	[9,22]
Gonioscopy	Closed angles, possible peripheral anterior synechiae	[15,20]
AS-OCT/UBM	Confirmed angle closure, ciliary body effusion	[27,32]
Axial Length/AC Depth	Short axial length (<22 mm), decreased chamber depth	[18,24]

Therapeutic interventions and outcomes

Immediate Medical Intervention Protocols

Prompt intervention in AACG, especially dengue-triggered AACG, is crucial to preserve vision and prevent permanent optic nerve damage. Acute management is based on a multimodal pharmacological approach to rapidly lower IOP. The first line of IOP reduction is typically an intravenous administration of mannitol, which works by osmotically drawing fluid from the vitreous body [15]. Oral acetazolamide, a carbonic anhydrase inhibitor, is also given simultaneously to decrease aqueous humor production, complementing the effect of mannitol. In addition to introducing topical anti-glaucoma medications - such as beta-blockers (e.g., timolol), alpha agonists (e.g., apraclonidine), and prostaglandin analogs - IOP is managed through multiple pharmacodynamic pathways.

Once instituted in a timely fashion, it provides important relief from symptoms like ocular pain, nausea, and blurred vision in as little as a few hours. Nevertheless, systemic variables such as fluid imbalance, capillary leak syndrome, and inflammatory mediators may influence the therapeutic response in dengue-associated AACG. Thus, systemic stabilization, interdisciplinary coordination, and ophthalmologic intervention are usually all necessary.

Neodymium-Doped Yttrium Aluminum Garnet (Nd:YAG) Peripheral Iridotomy: Description and Outcome Analysis

Once IOP is medically stable and corneal clarity improves, a definitive laser intervention - such as Nd:YAG peripheral iridotomy - can be performed. This procedure is intended to create a communication pathway between the anterior and posterior chambers of the eye to break the pupillary block and restore aqueous humor dynamics. Nd:YAG iridotomy is preferred due to its non-invasive nature and precise energy delivery, with minimal collateral tissue damage.

When dengue causes AACG, iridotomy has also been demonstrated to be successful when performed at the appropriate time. The formation of PAS can occur after delayed iridotomy and compromise the long-term patency of the angle [27]. There are reports that successful iridotomy results in a characteristic and prolonged reduction in IOP, resolution of corneal edema, and restoration of visual acuity [12].

While it is of benefit, complications such as iridotomy closure, transient IOP spikes, and bleeding can occur. Such risks can be minimized by pre-treatment with pilocarpine and post-laser steroids. In the first week, postoperative monitoring is critical to assess IOP control and to evaluate the functional status of the iridotomy opening [29].

Short-Term and Long-Term Clinical Outcomes

Treatment of dengue-associated AACG is generally associated with favorable short-term outcomes when initiated promptly. After combined medical and laser therapy, most patients usually experience a decrease in IOP to safe levels within 24 to 48 hours. Often, visual acuity returns and improves significantly with the restoration of corneal clarity and stability of optic nerve perfusion [33].

Delayed complications should be detected by follow-up evaluations at one week, one month, and three months. Serial tonometry, gonioscopy, and optic nerve imaging for monitoring signs of glaucoma progression are all part of long-term monitoring. While most dengue-triggered AACG cases do not progress to chronic glaucoma, anatomical predispositions and secondary mechanisms must be considered.

Lens status is an important factor in long-term management. Clear lens extraction has been advocated in phakic patients with persistent angle closure despite iridotomy, to deepen the anterior chamber and reduce angle crowding. Systemic recovery from dengue infection is also a determinant of ocular healing, especially in patients who develop postinfectious uveitis or choroidal effusion.

Timely pharmacologic intervention, laser therapy, and systemic disease management, in concert with an overall multidisciplinary approach, make a major impact on visual prognosis in affected individuals. Therefore, comprehensive care includes, but is not limited to, adherence to follow-up protocols, proper documentation, and patient education.

Methodological considerations and limitations

This review provides valuable insight into the very rare association of AACG occurring in the setting of dengue fever, and we emphasize the methodological limitations of single-case analyses. Although case reports are clinically illustrative, they are not generalizable and lack the statistical robustness to establish a causative relationship [12]. Additionally, this analysis does not include longitudinal follow-up data, which is necessary to assess the long-term ocular outcomes, recurrence risk, or progression to chronic glaucoma in post-dengue patients [19]. A second limitation is that, in all cases, it is impossible to completely discriminate dengue-induced AACG from dengue-related, practically idiopathic, or drug-induced AACG. Most importantly, overlapping clinical features of elevated IOP, corneal edema, and shallow anterior chamber can complicate diagnosis without adjunctive imaging and detailed systemic evaluation [18]. It is further complicated by systemic variables such as hydration status, concurrent medications, and inflammatory response profiles. Therefore, further studies with a larger sample size and standardized diagnostic protocols to validate the observed associations and to further explore the pathophysiologic mechanisms are warranted.

Future directions

Future research should focus on expanding such an evidence base to include multi-center observational studies and large case series, given the rarity and clinical complexity of AACG as a complication of dengue fever. Collaborative research of this type can assist in building epidemiological patterns, identifying at-risk populations, and increasing diagnostic specificity [4,21]. Subgroup analyses by age, comorbidities, and dengue severity will also be possible from large-scale data collection. However, more research is needed to understand the long-term course of chronic complications of dengue, such as visual field preservation, optic nerve health, and IOP stability. This will also allow for extended follow-up to determine the risk of recurrence and the potential transition to chronic glaucoma, which is poorly documented in the existing literature [17]. Furthermore, preventive techniques must be developed, particularly in places where dengue is prevalent. Ocular screening techniques should be incorporated into public health guidelines for dengue patients, especially those who have visual impairments. More simple, early recognition tools, like portable tonometry and point-of-care AS-OCT, would make a big difference. By establishing standardized diagnostic criteria and intervention algorithms, management would become more efficient, and their vision-related morbidity could be reduced.

## Conclusions

This paper underscores a significant but often underrecognized correlation between dengue fever and AACG, and describes how systemic infections can lead to emergent ophthalmic conditions. The clinical synthesis of findings shows that dengue-induced AACG is an inflammatory, vascular, and anatomical disruption that requires timely and precise diagnosis. Visual acuity, IOP, and anterior segment imaging all need to be assessed as early ophthalmologic evaluations to identify angle closure and prompt appropriate medical or surgical interventions. In dengue-endemic regions, clinicians should be alert to ocular complaints in febrile patients with photophobia, pain, or blurred vision. Standardized diagnostic protocols, integrated with interdisciplinary management and patient education, can prevent vision loss and improve long-term outcomes. As this presentation is rare, healthcare providers should also document and report similar cases to help build a broader understanding of its pathogenesis. The cornerstone of managing this rare but vision-threatening complication of dengue infection is proactive awareness and early intervention.
